# Fetal Outcomes in Pregnancies Complicated by Intrahepatic Cholestasis of Pregnancy in a Northern California Cohort

**DOI:** 10.1371/journal.pone.0028343

**Published:** 2012-03-05

**Authors:** Michelle Rook, Juan Vargas, Aaron Caughey, Peter Bacchetti, Philip Rosenthal, Laura Bull

**Affiliations:** 1 Division of Pediatric Gastroenterology, Hepatology and Nutrition, Department of Pediatrics, University of California San Francisco, San Francisco, California, United States of America; 2 Department of Obstetrics, Gynecology and Reproductive Sciences, University of California San Francisco, San Francisco, California, United States of America; 3 Department of Radiology, University of California San Francisco, San Francisco, California, United States of America; 4 Department of Epidemiology and Biostatistics, University of California San Francisco, San Francisco, California, United States of America; 5 Department of Surgery, University of California San Francisco, San Francisco, California, United States of America; 6 Liver Center Laboratory and Institute for Human Genetics, Department of Medicine, University of California San Francisco, San Francisco, California, United States of America; The University of Adelaide, Australia

## Abstract

**Background:**

Intrahepatic cholestasis of pregnancy (ICP) has important fetal implications. There is increased risk for poor fetal outcomes, including preterm delivery, meconium staining of amniotic fluid, respiratory distress, fetal distress and demise.

**Methods:**

One hundred and one women diagnosed with ICP between January 2005 and March 2009 at San Francisco General Hospital were included in this study. Single predictor logistic regression models were used to assess the associations of maternal clinical and biochemical predictors with fetal complications. Clinical predictors analyzed included age, race/ethnicity, gravidity, parity, history of liver or biliary disease, history of ICP in previous pregnancies, and induction. Biochemical predictors analyzed included serum aspartate aminotransferase, alanine aminotransferase, alkaline phosphatase, total bilirubin, direct bilirubin, albumin, total protein, and total bile acids (TBA).

**Results:**

The prevalence of ICP was 1.9%. Most were Latina (90%). Labor was induced in the majority (87%) and most were delivered by normal spontaneous vaginal delivery (84%). Fetal complications occurred in 33% of the deliveries, with respiratory distress accounting for the majority of complications. There were no statistically significant clinical or biochemical predictors associated with an increased risk of fetal complications. Elevated TBA had little association with fetal complications until reaching greater than 100 µmoL/L, with 3 out of 5 having reported complications. ICP in previous pregnancies was associated with decreased risk of fetal complications (OR 0.21, p = 0.046). There were no cases of late term fetal demise.

**Conclusions:**

Maternal clinical and laboratory features, including elevated TBA, did not appear to be substantial predictors of fetal complications in ICP.

## Introduction

Intrahepatic cholestasis of pregnancy (ICP) is the most common liver disease seen in pregnancy. It is typically a reversible cholestatic disease presenting in the second to third trimester of pregnancy and is characterized by pruritus predominantly of the palms and soles, elevated serum aminotransferases and/or elevated serum bile acid levels (>or = 10micromol/L) with spontaneous relief of laboratory abnormalities and symptoms promptly after delivery but no later than one month post partum.

The incidence of ICP has been found to be variable, with 0.1–1.5% of the population of Central and Western Europe and North America, and 1.5–4% in Chile and Bolivia [1]. There is minimal data on the incidence of ICP in the United States, with recent reports stating an incidence of 0.3%, and a recent study on a Latina population in Southern California that determined the overall prevalence in their population to be 5.6%, 10 to 100 times higher than previously reported for the U.S. population [2]–[4]. It is possible that the low incidence and prevalence rates in the United States may be due to underdiagnosis.

The pathogenesis of ICP, although not well defined, is thought to be multifactorial, including an environmental, genetic and hormonal basis for disease [5]. ICP is relatively benign to women, but it has been reported to have important fetal implications. ICP has been found to be associated with increased risk of preterm delivery, meconium staining of amniotic fluid, fetal bradycardia, fetal distress and fetal demise [2], [5], [6]. The underlying mechanisms associated with poor fetal outcome are largely unknown. Poor fetal outcomes, including asphyxial events and spontaneous preterm delivery, have been shown to be associated with elevated maternal total serum bile acids (>40 micromol/L) in pregnancy [7]; however, specific predictors of pregnancy outcomes have not been consistently identified [6], [7]. Maternal treatment with ursodeoxycholic acid (UDCA), which provides symptomatic relief of pruritus, has been shown to yield a significant improvement on biochemical markers, and gestational age of delivery in patients with ICP [8], [9]. The use of UDCA may have important implications in pregnancy outcomes. The aim of our study is to describe maternal and fetal characteristics associated with ICP in a cohort of patients in Northern California and to determine if significant clinical and biochemical predictors of fetal complications in women diagnosed with ICP exist.

## Materials and Methods

### Ethics Statement

This retrospective study is included in the approved University of California San Francisco (UCSF) CHR (Committee on Human Research). To note, the CHR is the name of UCSF's IRB. The study was approved by the CHR. Informed consent was not required as it was a retrospective study and study subjects were de-identified.

### Patient Population

One hundred and one women diagnosed with clinical ICP were included in this retrospective study. Patients were identified as having clinical ICP, if they presented to their obstetric provider in their second or third trimester of pregnancy with clinical symptoms of pruritus that could not be defined by any other etiology. Inclusion criteria for patient selection included: being diagnosed with ICP by a physician or certified nurse midwife (CNM), having appropriate documentation in medical records, and development of pruritus during the second or third trimester of pregnancy. All subjects had ICD 9 codes consistent with disorders of biliary ducts at the time of delivery. Exclusion criteria for the study included diagnosis of chronic liver disease (hepatitis B, hepatitis C, primary biliary cirrhosis, primary sclerosing cholangitis, symptomatic cholelithiasis, cholecystitis, Wilson disease, alpha-1-antitrypsin deficiency, cytomegalovirus, Epstein-Barr virus, or autoimmune hepatitis) that could not be differentiated from ICP at the time of delivery, acute fatty liver of pregnancy (AFLP) and HELLP syndrome (hemolysis, elevated liver enzymes, low platelets). Six women with hepatitis B or C were included in the study, as their presenting symptoms were consistent with ICP, rather than manifestations of infectious hepatitis. All patients were seen at the San Francisco General Hospital (SFGH), Department of Obstetrics and Gynecology, between January 1, 2005 and March 1, 2009.

### Measurements

After IRB approval was obtained, all women who had received a diagnosis of ICP during the study time period were identified. The subjects' medical records were abstracted and data on specific maternal and fetal characteristics was collected on each patient included in the study. The maternal data collected included maternal age at time of delivery, race/ethnicity, type of delivery, history of ICP in previous pregnancy, history of biliary or liver disease, spontaneous vs. induced labor, induction indications, gravidity, parity, weight (kg) and height (cm). BMI was calculated based on pre-pregnancy weight and height. The biochemical values evaluated included maximum serum aspartate aminotransferase (AST), alanine aminotransferase (ALT), total bilirubin (TB), direct bilirubin (DB), albumin (ALB), total protein (TP), alkaline phosphatase (ALP), total bile acids (TBA), cholic acid (cholic), deoxycholic acid (deoxy) and chenodeoxycholic acid (cheno) levels. Serum laboratory tests were typically obtained at the time of presentation, or at the time of delivery, and in cases where more than one laboratory test was performed, we used the maximum values measured for AST, ALT, TB, DB, ALP, TBA, cholic, deoxy and cheno, and the minimum values for ALB, and TP, as maximum and minimum values of these respective laboratory tests most likely correlate with hepatic dysfunction if abnormal. Serum biochemical and bile acid tests were determined using routine laboratory methods.

The primary outcome measured in this study was a composite outcome of one or more fetal or neonatal complications. The fetal complications included fetal distress, major congenital anomalies, hyperbilirubinemia, meconium staining of amniotic fluid at delivery, meconium aspiration, pneumonia, respiratory distress and sepsis. Fetal distress was defined by repetitive severe variable decelerations, or repetitive late decelerations, or fetal bradycardia of less than 110 beats per minute lasting 3 minutes or longer, requiring emergent delivery. Respiratory distress was defined as any neonate that required intubation, continuous positive airway pressure (CPAP), bag/mask ventilation post-partum, diagnosis of pneumonia, documentation of respiratory distress secondary to meconium aspiration, or documentation by a health care provider based on physical exam. Additional perinatal data collected included estimated gestational age at time of delivery, birth weight (grams), 1 minute Apgar score and 5 minute Apgar score. The clinical, biochemical and pregnancy outcomes were extracted for each patient by review of patient medical records with a data abstraction form and then entered into an excel spreadsheet to create the ICP database.

### Statistical Analysis

Descriptive statistics were used to characterize the cohort for our data analysis. The results are expressed as means +/− SD, or medians and interquartile ranges, as appropriate. Continuous predictor variables were dichotomized to binary variables for ease of interpretability. Advanced maternal age was defined as age greater than 35 years at delivery. The maximum values for AST, ALT, cholic, deoxy, and cheno were dichotomized into binary variables based on our laboratories standardized normal values. AST was considered high if values were greater than 40 IU/L, ALT was considered high if values were greater than 45 IU/L, cholic was considered high if values were greater than 3.1 µmoL/L, deoxy was considered high if values were greater than 7.3 µmoL/L, and cheno was considered high if values were greater than 9.9 µmoL/L. TBA was then categorized into four groups: less than 10 umoL/L, 10–40 umoL/L, 40–100 umoL/L, and greater than 100 Iu/L. The ‘less than 10 umoL/L’ was used as the reference group for comparison. Single-predictor logistic regression models were used to assess the unadjusted associations of clinical and biochemical predictors with fetal complications and respiratory distress. Two predictor models were generated, controlling for a maternal history of hepatic and biliary disease. Results were expressed as odds ratios. Statistical analysis was conducted in STATA version 10. (Stata Corp, College Station, TX). All reported p values are two-sided, and p<0.05 was considered statistically significant.

## Results

ICP was diagnosed in 101/5,238 pregnant women from January 1, 2005 to March 1, 2009, for a prevalence of 1.9%. Table 1 displays the maternal demographic features of the ICP population. The mean maternal age at time of delivery was 27.5 years (S.D. ±5.8) with a range of 16 to 41 years. Most of the ICP population was Latina (90%), whereas in the overall population during the study period 63% of pregnant women were Latina. Delivery methods in women with ICP included normal spontaneous vaginal delivery (84%), low transverse cesarean section (14.9%), and one patient had a vacuum assisted delivery. Delivery methods in the overall population included normal spontaneous vaginal delivery (74%), vacuum assisted delivery (3.5%), forceps delivery (2%) and low transverse cesarean section (21%). A history of ICP in previous pregnancies existed in 20 of the ICP patients, and 16 women had a history of other biliary or hepatic disease (Table 2). Medical histories were significant for cholelithiasis in five patients, cholecystitis in two patients, cholecystectomy in three patients, hepatitis A in one patient, hepatitis B in three patients, and hepatitis C in three patients. It was deemed by documentation that none of the patients had active disease at the time of presentation with ICP-associated symptoms. There were also maternal histories of post-partum hemorrhage, spontaneous abortion, peri-natal fetal death, and postnatal infant death in previous pregnancies (Table 2). Most of the patients in the ICP cohort were induced (87%), with diagnosis of ICP being the predominant indication (95.5%). Other induction indications included premature rupture of membranes (PROM), diabetes, oligohydramnios, and pre-eclampsia. The average pre-pregnancy BMI was calculated to be 27.7 (S.D. ±6.1). All patients presented with pruritus during the second or third trimester, and no patients were found to be jaundiced. UDCA was administered to 29% of the patients for treatment of pruritus.

**Table 1:** Maternal Demographics.

	N = 101 +/− S.D.
Age (years)	27.5+/−5.8
Race/ethnicity	
Latina	91
Asian	3
Black	4
White	2
Unknown	1
Delivery Methods	
Vaginal	86
Cesarian section	15
History ICP	20
Liver/Biliary History	16
Induction	88
Induction Indications	
ICP	84
PROM	1
Diabetes	1
Oligohydramnios	1
Preeclampsia	3
Gravida n = 98	3+/−1.4
Parity n = 98	1+/−1.2
BMI n = 76	27.7+/−6.1

S.D. = standard deviation, BMI = body mass index.

**Table 2:** Maternal Medical History.

	N = 101
Cholecystitis	2
Cholecystectomy	3
Cholelithiasis	5
Chorioamnionitis	1
Hepatitis A	1
Hepatitis B	3
Hepatitis C	3
Postpartum Hemorrhage	2
Spontaneous Abortion	2
*Perinatal Death	1
*Postnatal Death	1

Perinatal death = stillbirths or death within 1^st^ week of life.

Postnatal death = death after 1^st^ week of life.

Table 3 describes the median maximum laboratory values in the ICP cohort prior to delivery. Total bile acids were measured in 72% of the patients. The median maximum TBA was 23.4, with an interquartile range of 10.6 to 42. Elevated TBA greater than 10 µmoL/L were present in 75% of the patients that had TBA sent. Cholic acid, deoxycholic acid and chenodeoxycholic acid were measured in 72 patients. Hepatitis A serology was assayed in 45 patients; 43 patients had non-reactive titers. Hepatitis B surface antigen was assayed in 99 patients; 97 patients had non-reactive titers. Hepatitis B core antibody titers were obtained in 50 patients; 47 patients had non-reactive titers. Hepatitis C antibody titers were assayed in 55 patients; 52 patients had non-reactive titers.

**Table 3:** Maternal Laboratory Values*.

	N	Median	Interquartile Range
AST (U/L)	99	44	30–89
ALT (U/L)	99	62	33–139
ALP (U/L)	71	251	192–329
TB (mg/dL)	81	0.5	0.4–0.8
DB (mg/dL)	77	0.2	0.1–0.3
ALB (g/dL)	73	3.5	3.4–3.7
TP (g/dL)	74	6.5	6.0–6.7
TBA (µmol/L)	73	23.4	10.6–42
Cholic (µmol/L)	72	12	3.8–25.0
Deoxy (µmol/L)	72	2.5	1.7–4.3
Cheno (µmol/L)	72	6.1	3.0–13.9

*based on maximum value recorded.

U = units, L = liter, mg = milligram, dL = deciliter, µmol = micromole.

Neonatal characteristics at the time of delivery are described in Table 4. Mean estimated gestational age was 37 weeks (S.D. ±1.2) and mean birth weight was 3,126.3 grams (S.D. ±519.1). Mean one minute and five minute Apgar scores were 8 (S.D. ±1) and 9 (S.D. ±1). There were no 5 minute Apgar scores less than 7. In the overall population of pregnant women, 3.8% of deliveries had a 5 minute Apgar score less than 7. Fetal complications occurred in 33% of the deliveries in the ICP cohort. The most common perinatal complications were respiratory distress, meconium staining of the amniotic fluid, and fetal distress. Fetal demise did not occur in any of the patients. Fetal complication occurred in 5 out of 18 patients in the TBA <10 group (28%), 10 out of 34 patients in the TBA 10–40 group (29%), 3 out of 16 in the TBA 40–100 group (19%) and 3 out of 5 in the TBA >100 group (60%).

**Table 4:** Fetal Characteristics at Birth.

	N = 101 +/− S.D.
EGA (weeks)	37+/−1.2
Birthweight (grams)	3126.3+/−519.1
1 minute APGAR	8+/−1
5 minute APGAR	9+/−1
Complications	33
Respiratory Distress	17
Meconium	9
Fetal Distress	5
Congenital Anomalies	2
Hyperbilirubinemia	2
Sepsis	2
Pneumonia	1

S.D. = standard deviation.

Predictors of fetal complications were evaluated by logistic regression and described in Table 5. Advanced maternal age may have a minor impact on risk, but the uncertainty around this is very wide. Latina race appeared to have a substantial protective effect, but uncertainty around this result was again very great and it did not reach statistical significance. Maternal history of hepatic or biliary disease not including ICP, although not statistically significant, appeared to be associated with higher risk of fetal complications (OR 2.10, 95% CI 0.70, 6.28). A maternal history of having ICP in a previous pregnancy was found to be associated with an 80% decreased odds of having a fetal complication in the current pregnancy (OR 0.21, p = 0.046). Elevated maternal laboratory values (AST, ALT) during pregnancy did not correlate substantially with increased odds of fetal complications. TBA>10 µmol/L were not found to be statistically significant predictors of fetal complications. This study suggests that TBA>100 may indicate substantially increased risk, but the confidence interval is too wide to provide strong evidence for this. The use of ursodiol to treat pruritus during pregnancy was not associated with a decreased risk of fetal complications (OR 1.34, p = 0.53). Twenty nine patients were treated with UDCA. The median TBA for those who received UDCA was 23.4 (interquartile range 12.8 to 31.7) and the median TBA for those who did not receive UDCA was 21.9 (interquartile range 8.3 to 42); this difference was not statistically significant (p = 0.57, Mann-Whitney test). Four out of five patients with TBA>100 were treated with ursodiol. Those patients who were induced may have an increased risk of fetal complications (OR 2.57, p = 0.24). The number of previous pregnancies and previous births were not statistically significant predictors of fetal complications; however, it does appear that there is a trend towards reduced risk with higher values (gravidity OR 0.77, p = 0.15; parity 0.74, p = 0.16). No qualitative differences were found when controlling for maternal history of hepatic or biliary disease in a two predictor models.

**Table 5:** Predictors of Fetal Complications.

	OR	P	95% CI
Advanced Maternal Age	1.20	0.80	0.28–5.17
Latina	0.38	0.15	0.10–1.42
Historyliver/biliary disease	2.10	0.19	0.70–6.28
ICP in previous pregnancy	0.21	0.05	0.05–0.97
Elevated ALT	0.82	0.66	0.34–1.97
Elevated AST	0.76	0.53	0.32–1.80
TBA (reference group <10)			
TBA 10–40	1.08	0.90	0.30–3.85
TBA 40–100	0.60	0.54	0.12–3.05
TBA>100	3.90	0.20	0.49–30.76
Ursodiol use	1.34	0.53	0.53–3.38
Gravida	0.77	0.15	0.54–1.10
Parity	0.74	0.16	0.49–1.13
Induction	2.57	0.24	0.53–12.36

## Discussion

The data on ICP in the United States is extremely limited despite its high frequency. The prevalence of ICP in this retrospective cohort of patients was found to be 1.9%, similar to other data from Central and Western Europe. Previous literature reports 0.3% prevalence in the United States [4]. The prevalence is higher in Chile and Bolivia, with rates of 1.5–4%, and 9% [1], [10]. In our study, in which women with ICP were identified from an ethnically/racially mixed study population, Latina women had a greater likelihood of having ICP than other women (RR 4.96, 95%CI 2.59, 9.51, p<0.0001), suggesting that genetic factors may play a role in the development of ICP. In a recent study of a largely Latina Los Angeles population, the overall prevalence of ICP was found to be 5.6% [3].

Although ICP is usually relatively benign to the mother, it is known that the risk of fetal complications is increased in pregnancies affected by ICP. These include increased risks of meconium stained amniotic fluid, preterm delivery, fetal distress and intrauterine fetal demise (IUFD). In this study, we found that 33% of the births had an associated perinatal complication including respiratory distress syndrome, meconium staining of amniotic fluid, fetal distress, congenital anomalies, sepsis, hyperbilirubinemia and pneumonia. There were no episodes of IUFD. Previous studies have reported meconium staining in 24%, and intrauterine fetal demise in 0.4% of ICP cohorts [7]. The incidence of meconium staining of amniotic fluid at full term in a normal pregnant population varies between 17–24% and at 37 weeks gestational age is ∼5%, compared to the 9% incidence observed in our study [11], [12]. Respiratory distress accounted for 52% of the complications observed post-partum.

It has been reported that the incidence of respiratory distress syndrome in neonates born to mothers with ICP is twice that of the normal population [13], [14]. This can be due in part to the earlier gestational age at delivery, but neonatal respiratory distress syndrome has been demonstrated to be associated with ICP, based on analysis of bronchoalveolar lavage fluid of neonates born to mothers with ICP [14]. Specifically, it has been hypothesized that bile acids can produce surfactant depletion in the alveoli [13], [14]. Animal models have shown microscopic findings of atelectasis, poling of eosinophilic substances in intra-alveolar spaces, and formation of hyaline membranes in rabbits after intratracheal instillation of taurocholic acid [15]. When treated with surfactant, alveoli responded appropriately, and had increased aeration [15]. In another study, bronchial alveolar lavage was performed during necropsy on 12 infants and it was found that there were lower phospholipid levels, and higher bile acid levels [16]. This suggests a possible interaction between bile acids and surfactant mediated through the phospholipase A2, which synthesizes surfactant in the lung [16]. More details are necessary to determine the effects of bile acids on fetal lung maturity, surfactant production, and respiratory distress.

The risk of fetal morbidity and mortality in ICP is higher than in the general population [7], [17]–[20]. Even though in this study there were no cases of IUFD, the overall complication rate is still concerning. Current management of ICP is induction of labor at 36–38 weeks gestational age, regardless of TBA levels; however, it has been suggested that expectant management can be considered for those with TBA<40 µmol/L [7]. A study by Glantz et al concluded that pregnancies in women with TBA>40 µmol/L had an increased fetal risk of preterm delivery, asphyxia events, meconium staining of amniotic fluid, and green-staining of placenta and membranes, while those with TBA between 10–39 µmol/L had minimal to no increased risk compared to women with pruritus but normal TBA, and that based on these findings the latter women could be managed expectantly, and possibly reduce the costs of medical care. [7] It did not, however, provide the odds ratios for these direct comparisons, so the comparability of their results to ours is unclear. In our study, there were no statistically significant clinical or biochemical predictors associated with an increased risk of fetal complications. This may be in part due to differences in clinical practice, where we typically deliver patients with identified ICP at ∼37 weeks gestational age, where as in the Glantz et al. study there were no specific instructions given to obstetricians on how to manage the pregnancies or timing of delivery. This may also explain the differences seen in fetal demise, as the majority of cases of IUFD occur late in gestation, usually after 36 weeks of gestation. In our study, only prior history of ICP showed significance, and this was an association with a decreased rate of fetal complication. A history of hepatic/biliary disease, although not statistically significant, may have an important association with increased fetal complications. Those with hepatic or biliary disease may be at increased risk for fetal complications. A maternal history of ICP in a previous pregnancy was estimated to decrease the risk of complication by 80%, suggesting an increase in awareness of ICP in the patient and provider, with possibly increased fetal monitoring, and earlier maternal obstetric care, which can decrease the chance of fetal complications. The mean gestational age in those with a history of ICP was 37 weeks (range 36–39), and the mean gestational age in those that did not have ICP in previous pregnancies was also 37 weeks (range 33–40). The proportion of deliveries with a gestational age greater than 37 weeks was 35% in those with a history of ICP and 26% in those without a history of ICP.

Current pharmacologic treatment includes the use of UDCA upon diagnosis of ICP. UDCA has been shown to have greater efficacy than other treatment modalities, including the use of S-adenosyl-L-methionine, dexamethasone, and cholestyramine, regarding maternal pruritus, and improving TBA and serum transaminases. In a randomized, double-blinded placebo controlled trial by Glantz et al., it was found that UDCA was more effective than dexamethasone in relieving pruritus, and improving serum biochemical markers of ICP, however there was no effect of UDCA or dexamethasone on fetal complication rates. [21] In our study, there was no clinically significant effect of UDCA on fetal complications (Table 5).

TBA was categorized into four separate groups: normal or less than 10 µmol/L, 10–40 µmol/L, 40–100 µmol/L, and >100 µmol/L. When compared to the reference group of normal (<10), there was no increase in fetal complications in the TBA 10–40 group or TBA 40–100 group, suggesting that elevated TBA up to 100 may not be particularly useful in determining the risk of fetal complications. When TBA was greater than 100 µmoL/L, 60% had reported complications, for an OR 3.90 but with a wide confidence interval (95% CI 0.49, 30.76).

Our study had several limitations. In a retrospective study, it is difficult to control for missing data and uniformity of laboratory studies ordered within a cohort. The majority of the patients did have TBA, AST, and ALT sent. However, not all women were tested for other causes of potential liver disease. Total serum bile acids typically peak 30–90 minutes after meals, and there was no documentation of whether the TBA levels sent were fasting levels. Most likely, they were sent as part of routine laboratory testing done at the time of the clinic visit, or when the patient was admitted for delivery. The criteria for diagnosing ICP are also subjective, as there are no current uniform criteria for the diagnosis of ICP. Most studies use elevated serum bile acids or serum transaminase levels combined with pruritus during pregnancy; however, the serum bile acids may not necessarily be elevated at the time of a blood draw due to fasting versus non fasting state and there may be a greater rise towards the later weeks of pregnancy. In addition, the turnaround time for receiving the laboratory results may be 1–2 weeks, making it difficult to await the result when deciding on induction of labor to reduce potential fetal complications, specifically fetal demise.

In summary, in this cohort of ICP patients, 33% of the pregnancies resulted in perinatal complications, with respiratory distress being the most common complication. A history of liver and biliary disease and TBA greater than 100 may be clinically relevant, but our data were not conclusive. Our results suggest that it may be difficult to use maternal clinical and biochemical features to accurately predict fetal complications.
